# Automation of the control system for drying grain crops of the technological process for obtaining biodiesel fuels

**DOI:** 10.1038/s41598-023-41962-0

**Published:** 2023-09-11

**Authors:** Anzhelika M. Eremeeva, Yury V. Ilyushin

**Affiliations:** 1 Department of Geoecology, Saint Petersburg Mining University, Saint Petersburg, Russia; 2 Faculty of Economics, Saint Petersburg Mining University, Saint Petersburg, Russia

**Keywords:** Information technology, Pollution remediation, Plant biotechnology, Chemical engineering, Chemical engineering, Biofuels

## Abstract

Process of grain drying is discussed by the authors, which is considered one of the preliminary stages in the technology of biodiesel production. The drying process has a number of disadvantages that affect the quality and cost of biodiesel fuel. The impossibility of uniform heating and maintaining the required temperature with minimal energy costs is considered one of the most important defects that deserve scientific research. The authors propose a method for changing the heating system and preheating raw materials, based on world experience. We carried out mathematical calculations, provided the change in the temperature field of the drying chamber over time, and we also performed industrial experiment. Based on our results we determined the optimal number of heating sources of raw materials, taking into account the dimensions of the drying chamber. The authors propose a technical solution with which a uniform temperature field can be obtained in the drying chamber. Thus, the uniformity of the chamber heating will be increased, and large operating costs associated with leakage of oil from the grains will be disappeared.

## Introduction

The growth of industry causes problems with increasing of source of raw materials, because extractable resources are exhaustible^1,2^. According to experts, oil as a main source of raw materials for production of wide variety of products will be exhausted in the next 100 years^3,4^, therefore, it will lead to critical decreasing of production. The systematic changing to regenerated energy supplies can prevent an economic crisis and decrease consequences of reduction oil sources^5^. One of perspective direction of this changing is replacement diesel oil (or part of diesel oil) to biodiesel, which is can be created with plant raw materials^6–9^. It will allow to reduce amount of oil fuel and to redirect it to chemical industry needs.

Technological process of creating biodiesel oil can be divided into three stages: preparation of raw materials, getting of biodiesel oil and refinement it. Consider the first step in detail. The first one of the technological row includes transportation, storing and following expression of oils from plant seeds. The most important problem of this step is conservation of oils in seeds and prevention of its evaporation under the influence of heating the grains. The decision of this problem on all stages of transportation and grain storing is to accomplish control of temperature and humidity. Every step includes different ways to do it: transportation includes close refrigerators with constant cooling, storage is furnished with system of climatic control in repositories, step of expression goes with defined technological conditions of regime. Maximum amount of dissipation takes place on a storage step. Permanent technical process, constant replenishment and extraction from repository of grain reserves, caking grain complicate to provide balance of temperature and humidity in storage rooms. Also, functioning of heating and cooling system in storage requires high energy costs which are connected with using of heating elements—tubular heaters. The use of temperature pre-set chambers partially solves this problem, but significantly increases the area of the granary. Despite of small production and technical limits this way is sufficiently expensive process. Alternative is stream loading system. In this way refrigerators load seed directly to spin system, therefore, percent of spin increases, but at the same time it increases logistic risks, which can causes full stop production in difficult climatic condition.

Consequently, searching of optimal technical solutions to ensure cost-effective grain storage is an important scientific-technical task to increase effectiveness of technical process biodiesel production.

## Existing technical solution

Biodiesel history started in 1900 when Rudolf Diesel demonstrated engine working on peanut oil on Paris world exhibition. Since then there are many scientific researches directed to upgrading technology of production and quality of fuel^10,11^. In particular, Islam S., Basumatary B., Rokhum S.L., Mochahari P.K., Basumatary S.^12,13^ and others consider using of heterogenous nanocatalysts to increase yield, but note necessity to strictly observe technical operating. Zambre A. conducts analysis of technological processes and together with group of researchers Upendran A., Shukla R., Chanda N., Katti K.K., Cutler C., Kannan R., Katti K.V. and others.^14,15^ conduct research on upgrading of getting biodiesel fuel technology. At the same time, authors note constant growth of biodiesel fuel production^16–18^ and state assumption necessity to divide big productions on small one because of logistic difficulties to transport raw materials and products. Athar M., Zaidi S., Inamuddin, Mohd Imran Ahamed, R. Boddula, M. Rezakazemi^19,20^ conduct analysis of types raw materials and technologies its preparation to get biodiesel. Brazilian scientist Ramos, L. P.; Silva, F. R.; Mangrich, A. S.; Cordeiro, C. S. Tecnologias^21^ conduct similar researching. Patil P. D., Madavi A., Bhusari P. S., Y. Zhang, M.A. Dube, D.D. McLean, M Kates^22^ consider process of secondary processing with getting of glycerin. Also, it is important to note works^23–28^ which show importance of temperature regimes and their influence on quality of getting products. Issues of heat exchange processes during storage and drying grain crops began ti be engaged long ago. These processes based on classic task of isothermal processes. The last works about this direction should be noted works authors Pavlov S.A., Frolova T.F.^29^, Ronzhin A.L., Savel'ev A.I.^30^, Sanford S., Go A.^31^, Bezyazychnyy V. F^32^. Also, it is important to note works about researching of thermal processes in close placement. Such works include the works of Alyabyev V.R., Bazhina T.P., Pershin I.M., Chernyshev A.B. and others^33–37^. In area of spatial accountiiong of heat exchanging processes, it is important to note work of Schäfer J.C., W.L. Martin, W.L. Martin, Partha S. Sarathi., McNab G., Lo H.^38^ Desoer C.A., Wing J.^39^, Martirosyan A.V.^40^, Kuharova T.V.^41^ and others^42^.

Thus, there is wide range of researchers connected with control drying plant work. However, as noted in most of the above studies, it is rather difficult to provide a continuous, uniform thermal field in view of the many dynamic parameters of the system. Using the terms of the theory of automatic control, it is necessary to note the complexity of modeling a thermodynamic system due to the multitude of chaotic dynamic influences.

It is necessarily to develop economically efficient system of control for temperature field in grain pre-storage chamber.

Stated task is achieved by accounting on mathematical model of chamber overall parameter, replacement of entire heating elements on spot worked in impulsive regime heaters.

## Methodology

Mathematical model of chamber represents thermal differential model of the following form:

1 $$\frac{{\partial Q\left( {x,y,z,t} \right)}}{\partial t} - a^{2} \left[ {\frac{{\partial^{2} Q\left( {x,y,z,t} \right)}}{{\partial x^{2} }} + \frac{{\partial^{2} Q\left( {x,y,z,t} \right)}}{{\partial y^{2} }} + \frac{{\partial^{2} Q\left( {x,y,z,t} \right)}}{{\partial z^{2} }}} \right] = f\left( {x,y,z,t} \right);$$

2 $$Q\left( {x,y,z,0} \right) = Q_{0} \left( {x,y,z} \right);$$

3 $$Q\left( {0,y,z,t} \right) = q_{1} \left( {y,z,t} \right); \, Q\left( {l_{1} ,y,z,t} \right) = q_{2} \left( {y,z,t} \right); \, Q\left( {x,0,z,t} \right) = q_{3} \left( {y,z,t} \right);$$

4 $$Q\left( {x,l_{2} ,z,t} \right) = q_{4} \left( {x,z,t} \right); \, Q\left( {x,y,0,t} \right) = q_{5} \left( {x,y,t} \right); \, Q\left( {x,y,l_{3} ,t} \right) = q_{6} \left( {x,y,t} \right);$$

5 $$0 \le x \le l_{1} ; \, 0 \le y \le l_{2} ; \, 0 \le z \le l_{3} ; \, t \ge 0; \, a > 0$$

In the given mathtmatical model Q is marked as a measered parameter (in our case, the temperature value), x, y, z—is a location of sensor of the heating element. F—is a output function. Equations (2–5) show boundary conditions of the object. The second equation show, that temperature is the same in all points of the investigated room in the initial moment of time. Equations (3–4) show process of temperature spread from the upper part of room to the lower part. Equation number 5 show area of invetigated parameters, for example, points x, y, z are inside an object which is limitid by coordinates from 0 to l1, l2, l3. Entering influence can be presented as point source which is described by next equation:

6 $$\begin{aligned} G\left( {x,y,z,\rho ,\nu ,\vartheta ,t} \right) & = \frac{8}{{l_{1} l_{2} l_{3} }}\sum\limits_{k,m,n = 1}^{\infty } {\exp \left[ { - a^{2} \pi^{2} t\left( {\frac{{k^{2} }}{{l_{1}^{2} }} + \frac{{m^{2} }}{{l_{2}^{2} }} + \frac{{n^{2} }}{{l_{3}^{2} }}} \right)} \right]} \sin \left( {\frac{k \cdot \pi \cdot x}{{l_{1} }}} \right) \\ & \quad \times \sin \left( {\frac{m \cdot \pi \cdot y}{{l_{2} }}} \right) \times \sin \left( {\frac{n \cdot \pi \cdot z}{{l_{3} }}} \right) \times \sin \left( {\frac{k \cdot \pi \cdot \rho }{{l_{1} }}} \right) \times \sin \left( {\frac{m \cdot \pi \cdot \nu }{{l_{2} }}} \right) \times \sin \left( {\frac{n \cdot \pi \cdot \vartheta }{{l_{3} }}} \right). \\ \end{aligned}$$

Green's function equation G determine temperature in point with coordinate x, y, z at point of time t. The object itself is characterized by the coefficient of temperature conductivity *a* and geometric parameter l_1_, l_2_, l_3_. The point of application of the influence of unit power is described by coordinates ρ, v, ϑ, where к, *т*, n are assigned. Mentioned heating elements, spaced at equal distances from each other, influence not only themselves, but also next heating elements. So, one heating element will have the maximum effect on the nearest adjacent heating element.

7 $$\begin{aligned} G\left( {x_{0} ,y_{0} ,z_{0} ,\rho ,\nu ,\vartheta ,t} \right) & = \frac{8}{{l_{1} l_{2} l_{3} }}\exp \left[ { - a^{2} \pi^{2} t\left( {\frac{{k^{2} }}{{l_{1}^{2} }} + \frac{{m^{2} }}{{l_{2}^{2} }} + \frac{{n^{2} }}{{l_{3}^{2} }}} \right)} \right]\sin \left( {\frac{k \cdot \pi \cdot x}{{l_{1} }}} \right) \\ & \quad \times \sin \left( {\frac{m \cdot \pi \cdot y}{{l_{2} }}} \right) \times \sin \left( {\frac{n \cdot \pi \cdot z}{{l_{3} }}} \right) \times \sin \left( {\frac{k \cdot \pi \cdot \rho }{{l_{1} }}} \right) \times \sin \left( {\frac{m \cdot \pi \cdot \nu }{{l_{2} }}} \right) \times \sin \left( {\frac{n \cdot \pi \cdot \vartheta }{{l_{3} }}} \right). \\ \end{aligned}$$

8 $$\begin{aligned} G\left( {x_{1} ,y_{0} ,z_{0} ,,\rho ,\nu ,\vartheta ,t} \right) & = \frac{8}{{l_{1} l_{2} l_{3} }}\exp \left[ { - a^{2} \pi^{2} t\left( {\frac{{k^{2} }}{{l_{1}^{2} }} + \frac{{m^{2} }}{{l_{2}^{2} }} + \frac{{n^{2} }}{{l_{3}^{2} }}} \right)} \right]\sin \left( {\frac{k \cdot \pi \cdot x}{{l_{1} }}} \right) \\ & \quad \times \sin \left( {\frac{m \cdot \pi \cdot y}{{l_{2} }}} \right) \times \sin \left( {\frac{n \cdot \pi \cdot z}{{l_{3} }}} \right) \times \sin \left( {\frac{k \cdot \pi \cdot \rho }{{l_{1} }}} \right) \times \sin \left( {\frac{m \cdot \pi \cdot \nu }{{l_{2} }}} \right) \times \sin \left( {\frac{n \cdot \pi \cdot \vartheta }{{l_{3} }}} \right). \\ \end{aligned}$$

9 $$\begin{aligned} G\left( {x_{0} ,y_{1} ,z_{0} ,,\rho ,\nu ,\vartheta ,t} \right) & = \frac{8}{{l_{1} l_{2} l_{3} }}\exp \left[ { - a^{2} \pi^{2} t\left( {\frac{{k^{2} }}{{l_{1}^{2} }} + \frac{{m^{2} }}{{l_{2}^{2} }} + \frac{{n^{2} }}{{l_{3}^{2} }}} \right)} \right]\sin \left( {\frac{k \cdot \pi \cdot x}{{l_{1} }}} \right) \\ & \quad \times \sin \left( {\frac{m \cdot \pi \cdot y}{{l_{2} }}} \right) \times \sin \left( {\frac{n \cdot \pi \cdot z}{{l_{3} }}} \right) \times \sin \left( {\frac{k \cdot \pi \cdot \rho }{{l_{1} }}} \right) \times \sin \left( {\frac{m \cdot \pi \cdot \nu }{{l_{2} }}} \right) \times \sin \left( {\frac{n \cdot \pi \cdot \vartheta }{{l_{3} }}} \right). \\ \end{aligned}$$

10 $$\begin{aligned} G\left( {x_{0} ,y_{0} ,z_{1} ,,\rho ,\nu ,\vartheta ,t} \right) & = \frac{8}{{l_{1} l_{2} l_{3} }}\exp \left[ { - a^{2} \pi^{2} t\left( {\frac{{k^{2} }}{{l_{1}^{2} }} + \frac{{m^{2} }}{{l_{2}^{2} }} + \frac{{n^{2} }}{{l_{3}^{2} }}} \right)} \right]\sin \left( {\frac{k \cdot \pi \cdot x}{{l_{1} }}} \right) \\ & \quad \times \sin \left( {\frac{m \cdot \pi \cdot y}{{l_{2} }}} \right) \times \sin \left( {\frac{n \cdot \pi \cdot z}{{l_{3} }}} \right) \times \sin \left( {\frac{k \cdot \pi \cdot \rho }{{l_{1} }}} \right) \times \sin \left( {\frac{m \cdot \pi \cdot \nu }{{l_{2} }}} \right) \times \sin \left( {\frac{n \cdot \pi \cdot \vartheta }{{l_{3} }}} \right). \\ \end{aligned}$$

Based on the isotropy of the investigated environment, it is necessary to take into account the effect not only on the heating elements but also on the sensors.

11 $$\begin{aligned} G\left( {x_{0} ,y_{0} ,z_{0} ,,\rho ,\nu ,\vartheta ,t} \right) & = \frac{8}{{l_{1} l_{2} l_{3} }}\sum\limits_{k,m,n = 1}^{\infty } {\exp \left[ { - a^{2} \pi^{2} t\left( {\frac{{k^{2} }}{{l_{1}^{2} }} + \frac{{m^{2} }}{{l_{2}^{2} }} + \frac{{n^{2} }}{{l_{3}^{2} }}} \right)} \right]} \sin \left( {\frac{k \cdot \pi \cdot x}{{l_{1} }}} \right) \\ & \quad \times \sin \left( {\frac{m \cdot \pi \cdot y}{{l_{2} }}} \right) \times \sin \left( {\frac{n \cdot \pi \cdot z}{{l_{3} }}} \right) \times \sin \left( {\frac{k \cdot \pi \cdot \rho }{{l_{1} }}} \right) \times \sin \left( {\frac{m \cdot \pi \cdot \nu }{{l_{2} }}} \right) \times \sin \left( {\frac{n \cdot \pi \cdot \vartheta }{{l_{3} }}} \right). \\ \end{aligned}$$

12 $$\begin{aligned} G\left( {x_{1} ,y_{0} ,z_{0} ,,\rho ,\nu ,\vartheta ,t} \right) & = \frac{8}{{l_{1} l_{2} l_{3} }}\sum\limits_{k,m,n = 1}^{\infty } {\exp \left[ { - a^{2} \pi^{2} t\left( {\frac{{k^{2} }}{{l_{1}^{2} }} + \frac{{m^{2} }}{{l_{2}^{2} }} + \frac{{n^{2} }}{{l_{3}^{2} }}} \right)} \right]} \sin \left( {\frac{k \cdot \pi \cdot x}{{l_{1} }}} \right) \times \\ & \quad \times \sin \left( {\frac{m \cdot \pi \cdot y}{{l_{2} }}} \right) \times \sin \left( {\frac{n \cdot \pi \cdot z}{{l_{3} }}} \right) \times \sin \left( {\frac{k \cdot \pi \cdot \rho }{{l_{1} }}} \right) \times \sin \left( {\frac{m \cdot \pi \cdot \nu }{{l_{2} }}} \right) \times \sin \left( {\frac{n \cdot \pi \cdot \vartheta }{{l_{3} }}} \right). \\ \end{aligned}$$

13 $$\begin{aligned} G\left( {x_{0} ,y_{1} ,z_{0} ,,\rho ,\nu ,\vartheta ,t} \right) & = \frac{8}{{l_{1} l_{2} l_{3} }}\sum\limits_{k,m,n = 1}^{\infty } {\exp \left[ { - a^{2} \pi^{2} t\left( {\frac{{k^{2} }}{{l_{1}^{2} }} + \frac{{m^{2} }}{{l_{2}^{2} }} + \frac{{n^{2} }}{{l_{3}^{2} }}} \right)} \right]} \sin \left( {\frac{k \cdot \pi \cdot x}{{l_{1} }}} \right) \\ & \quad \times \sin \left( {\frac{m \cdot \pi \cdot y}{{l_{2} }}} \right) \times \sin \left( {\frac{n \cdot \pi \cdot z}{{l_{3} }}} \right) \times \sin \left( {\frac{k \cdot \pi \cdot \rho }{{l_{1} }}} \right) \times \sin \left( {\frac{m \cdot \pi \cdot \nu }{{l_{2} }}} \right) \times \sin \left( {\frac{n \cdot \pi \cdot \vartheta }{{l_{3} }}} \right). \\ \end{aligned}$$

**Figure 1:**
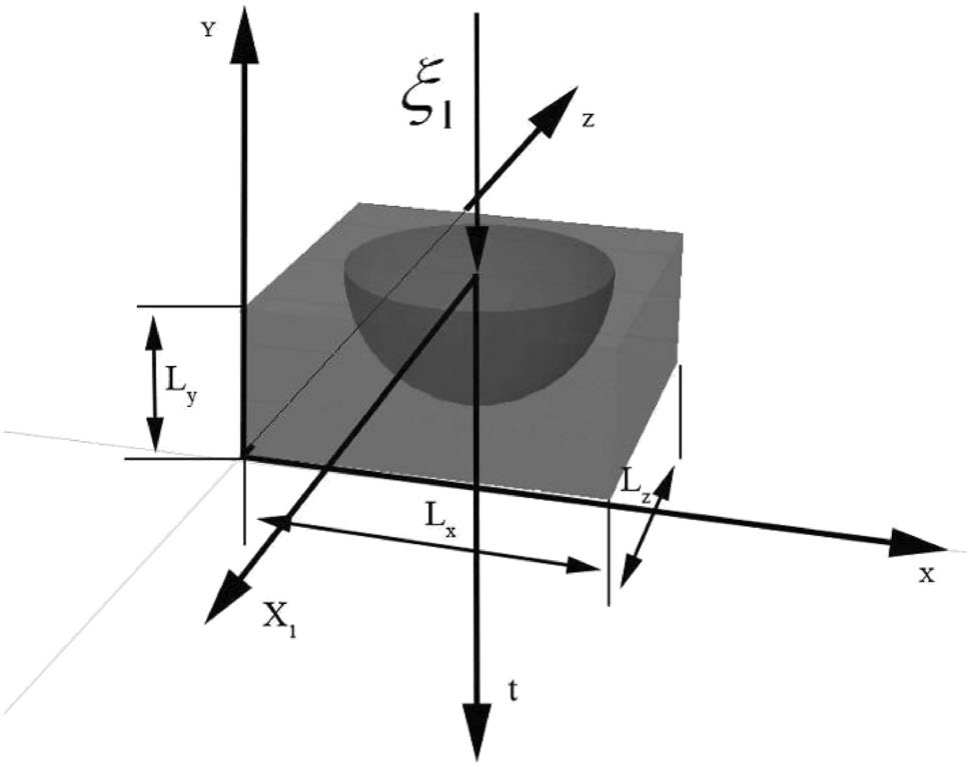
Graphical representation of the propagation of the initial pulse.

Thus, total temperature field influencing on all heating elements and sensor, will be expressed in next equation:

14 $$\begin{aligned} G\left( {x_{j} ,y_{j} ,z_{j} ,\rho ,\nu ,\vartheta ,t} \right) & = \sum\limits_{i = 1}^{d} {\frac{8}{{l_{1} \cdot l_{2} \cdot l_{3} }}} \sum\limits_{k,m,n = 1}^{\infty } {\sin \left( {\frac{{k \cdot \pi \cdot x_{j} }}{{l_{1} }}} \right) \times \sin \left( {\frac{{k \cdot \pi \cdot y_{j} }}{{l_{2} }}} \right)} \\ & \quad \times \sin \left( {\frac{{k \cdot \pi \cdot z_{j} }}{{l_{3} }}} \right) \times \sin \left( {\frac{{k \cdot \pi \cdot \rho_{i} }}{{l_{1} }}} \right) \times \sin \left( {\frac{{k \cdot \pi \cdot \nu_{i} }}{{l_{2} }}} \right) \times \sin \left( {\frac{{k \cdot \pi \cdot \vartheta_{i} }}{{l_{3} }}} \right) \\ & \quad \times \exp \left[ { - a^{2} \pi^{2} t\left( {\frac{{k^{2} }}{{l_{1}^{2} }} + \frac{{m^{2} }}{{l_{2}^{2} }} + \frac{{n^{2} }}{{l_{3}^{2} }}} \right)} \right] \times \sum\limits_{p} {\sum\limits_{k,m,n = 1}^{\infty } {\sin \left( {\frac{{k \cdot \pi \cdot x_{j} }}{{l_{1} }}} \right)} \times \sin \left( {\frac{{k \cdot \pi \cdot y_{j} }}{{l_{2} }}} \right)} \\ & \quad \times \sin \left( {\frac{{k \cdot \pi \cdot z_{j} }}{{l_{3} }}} \right) \times \sin \left( {\frac{{k \cdot \pi \cdot \rho_{z(p)} }}{{l_{1} }}} \right) \times \sin \left( {\frac{{k \cdot \pi \cdot \nu_{z(p)} }}{{l_{2} }}} \right) \times \sin \left( {\frac{{k \cdot \pi \cdot \vartheta_{z(p)} }}{{l_{3} }}} \right) \\ & \quad \times \exp \left[ { - a^{2} \pi^{2} \left( {t - \tau } \right)\left( {\frac{{k^{2} }}{{l_{1}^{2} }} + \frac{{m^{2} }}{{l_{2}^{2} }} + \frac{{n^{2} }}{{l_{3}^{2} }}} \right)} \right]. \\ \end{aligned}$$

where z(p) is the serial number of the heating element, d is a member of the Fourier series, τ—increment in time. Graphically, the distribution of temperature flows arising from the point exposure to the heating element is shown in the Fig. 1, where ξ_1_ is the coordinate of the heating element, L_x_, L_y_, L_z_ are the overall dimensions of the object, Z_1_ is the direction vector of the temperature field along the z axis, Y_1_ is the vector of the direction of the temperature field along the y axis.

**Figure 2:**
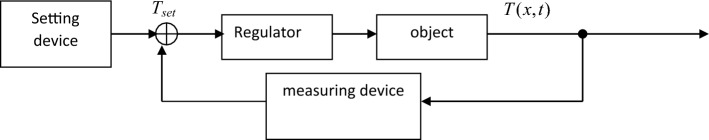
Block diagram of a closed control system.

When this system is closed into a closed spatially distributed system, in general, the resulting system will be presented in the form of a diagram in Fig. 2.

Two parameters are indicated in the block diagram: *T*—current temperature and *T*_*set*_—set temperature of the technological mode.

It is necessary to conduct experimental studies to analyze the stabilization of the temperature field in a given technological mode.

## Results

### Laboratory research

To do this, imagine the Green function to the Delphi language and write a program that model the behavior of the temperature field over time. Software code that implements this task will look like this:


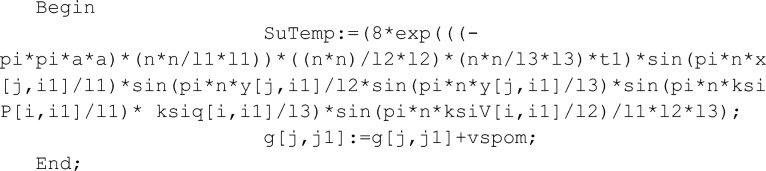


The fragment of the code for the placement of temperature sensors has the form:


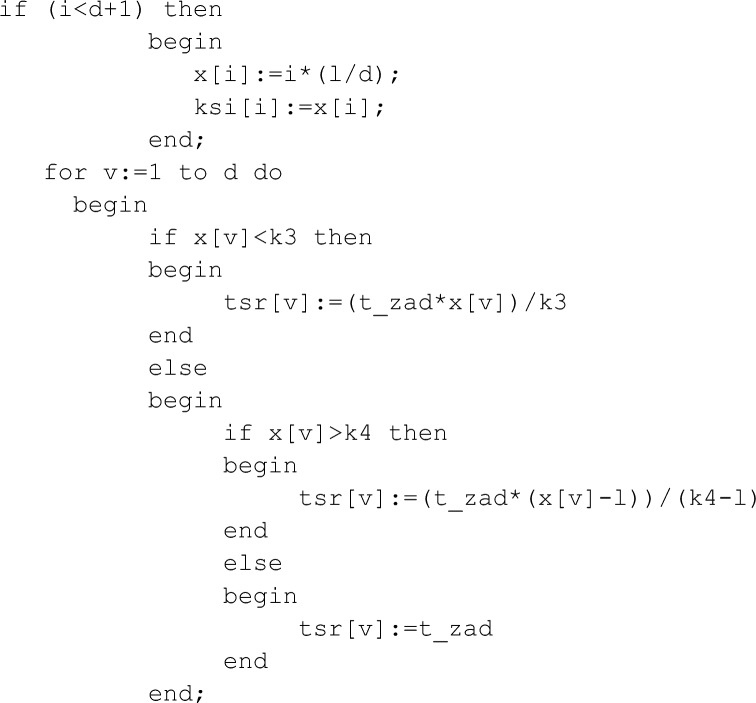


where temperature sensor is x[i] and heating element is ksi[i].

In the program code, the x[i] position is on the cross section with the ksi [i] position. This arrangement of the heating elements relative to their corresponding sensors allows you to record the values of the temperature field immediately after exposure to the pulse. However, the software package allows to place the temperature sensor at some distance from the heating source. If it is necessary, this function of the software algorithm will allow to measure the temperature in various parts of the rod.

Consider the different areas of the drying chambers. It is necessary to place the same number of heating elements in them. To increase the speed of the temperature set, we will increase the power of the heating elements. Let's set up a series of experiments on various metals and objects of various lengths (Table 1).

**Table 1:** Drying chamber area 5 m^2^.

*d* = 9	*d* = 8	*d* = 7	*d* = 6	*d* = 5
SuTemp[1,12] = 43,19	SuTemp[1,12] = 43,19	SuTemp[1,12] = 43,18	SuTemp[1,12] = 43,18	SuTemp[1,12] = 43,48
SuTemp[2,12] = 43,37	SuTemp[2,12] = 43,36	SuTemp[2,12] = 43,31	SuTemp[2,12] = 43,32	SuTemp[2,12] = 43,39
SuTemp[3,12] = 43,49	SuTemp[3,12] = 43,47	SuTemp[3,12] = 43,43	SuTemp[3,12] = 43,37	SuTemp[3,12] = 43,39
SuTemp[4,12] = 43,56	SuTemp[4,12] = 43,51	SuTemp[4,12] = 43,43	SuTemp[4,12] = 43,32	SuTemp[4,12] = 43,38
SuTemp[5,12] = 43,56	SuTemp[5,12] = 43,47	SuTemp[5,12] = 43,34	SuTemp[5,12] = 43,18	SuTemp[5,12] = 43,45
SuTemp[6,12] = 43,49	SuTemp[6,12] = 43,36	SuTemp[6,12] = 43,19	SuTemp[6,12] = 43,26	
SuTemp[7,12] = 43,37	SuTemp[7,12] = 43,19	SuTemp[7,12] = 43,42		
SuTemp[8,12] = 43,19	SuTemp[8,12] = 43,78			
SuTemp[9,12] = 43,14				

*d* = 14	*d* = 13	*d* = 12	*d* = 11	*d* = 10
SuTemp[1,12] = 43,20	SuTemp[1,12] = 43,20	SuTemp[1,12] = 43,19	SuTemp[1,12] = 43,19	SuTemp[1,12] = 43,19
SuTemp[2,12] = 43,39	SuTemp[2,12] = 43,38	SuTemp[2,12] = 43,38	SuTemp[2,12] = 43,38	SuTemp[2,12] = 43,37
SuTemp[3,12] = 43,56	SuTemp[3,12] = 43,55	SuTemp[3,12] = 43,54	SuTemp[3,12] = 43,53	SuTemp[3,12] = 43,51
SuTemp[4,12] = 43,70	SuTemp[4,12] = 43,68	SuTemp[4,12] = 43,66	SuTemp[4,12] = 43,64	SuTemp[4,12] = 43,60
SuTemp[5,12] = 43,80	SuTemp[5,12] = 43,77	SuTemp[5,12] = 43,74	SuTemp[5,12] = 43,69	SuTemp[5,12] = 43,63
SuTemp[6,12] = 43,87	SuTemp[6,12] = 43,82	SuTemp[6,12] = 43,76	SuTemp[6,12] = 43,69	SuTemp[6,12] = 43,60
SuTemp[7,12] = 43,89	SuTemp[7,12] = 43,82	SuTemp[7,12] = 43,74	SuTemp[7,12] = 43,64	SuTemp[7,12] = 43,51
SuTemp[8,12] = 43,87	SuTemp[8,12] = 43,77	SuTemp[8,12] = 43,66	SuTemp[8,12] = 43,53	SuTemp[8,12] = 43,37
SuTemp[9,12] = 43,80	SuTemp[9,12] = 43,68	SuTemp[9,12] = 43,54	SuTemp[9,12] = 43,38	SuTemp[9,12] = 43,19
SuTemp[10,12] = 43,70	SuTemp[10,12] = 43,55	SuTemp[10,12] = 43,38	SuTemp[10,12] = 43,19	SuTemp[10,12] = 43,50
SuTemp[11,12] = 43,56	SuTemp[11,12] = 43,38	SuTemp[11,12] = 43,19	SuTemp[11,12] = 43,85	
SuTemp[12,12] = 43,39	SuTemp[12,12] = 43,20	SuTemp[12,12] = 43,21		
SuTemp[13,12] = 43,20	SuTemp[13,12] = 43,56			
SuTemp[14,12] = 43,92				

SuTemp is a programming module development function of the form SuTemp[x,y] = z, which returns the location of the heating element x at a certain time interval y and the temperature value at this point z. SuTemp is measured in °C.

**Figure 3:**
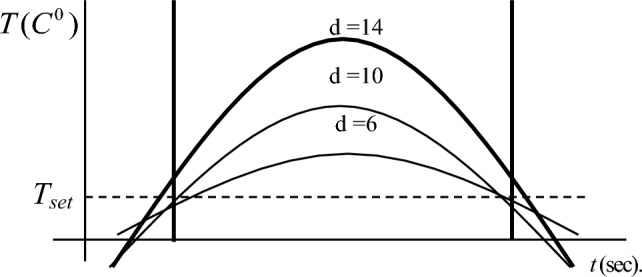
Temperature field values depending on time.

After analyzing the data in the table, we can conclude that the unjustified use of the maximum possible number of heating elements. If it is necessary to stabilize the temperature with an interval of 13.6°, then the installation of 10 heating elements is sufficient, while a larger number will be redundant (Fig. 3).

It is logical to assume that with an increase in the volume of the drying chamber, a greater number of heating elements will be involved. But it remains unclear whether the set temperature will be maintained. Let us carry out studies with a larger heating area, but with a fixed number of heating elements (Tables 2, 3, 4).

**Table 2:** Drying chamber area 10 m^2^.

*d* = 9	*d* = 8	*d* = 7	*d* = 6	*d* = 5
SuTemp[1,12] = 52,03	SuTemp[1,12] = 52,01	SuTemp[1,12] = 51,99	SuTemp[1,12] = 51,95	SuTemp[1,12] = 51,89
SuTemp[2,12] = 53,82	SuTemp[2,12] = 53,73	SuTemp[2,12] = 53,59	SuTemp[2,12] = 53,39	SuTemp[2,12] = 53,07
SuTemp[3,12] = 55,15	SuTemp[3,12] = 54,87	SuTemp[3,12] = 54,48	SuTemp[3,12] = 53,91	SuTemp[3,12] = 53,07
SuTemp[4,12] = 55,86	SuTemp[4,12] = 55,27	SuTemp[4,12] = 54,48	SuTemp[4,12] = 53,39	SuTemp[4,12] = 51,89
SuTemp[5,12] = 55,86	SuTemp[5,12] = 54,87	SuTemp[5,12] = 53,59	SuTemp[5,12] = 51,95	SuTemp[5,12] = 51,75
SuTemp[6,12] = 55,15	SuTemp[6,12] = 53,73	SuTemp[6,12] = 51,99	SuTemp[6,12] = 5[2,12]	
SuTemp[7,12] = 53,82	SuTemp[7,12] = 52,01	SuTemp[7,12] = 52,49		
SuTemp[8,12] = 52,03	SuTemp[8,12] = 52,86			
SuTemp[9,12] = 53,22				

*d* = 14	*d* = 13	*d* = 12	*d* = 11	*d* = 10
SuTemp[1,12] = 52,07	SuTemp[1,12] = 52,06	SuTemp[1,12] = 52,06	SuTemp[1,12] = 52,05	SuTemp[1,12] = 52,04
SuTemp[2,12] = 54,04	SuTemp[2,12] = 54,01	SuTemp[2,12] = 53,98	SuTemp[2,12] = 53,94	SuTemp[2,12] = 53,89
SuTemp[3,12] = 55,80	SuTemp[3,12] = 55,73	SuTemp[3,12] = 55,63	SuTemp[3,12] = 55,51	SuTemp[3,12] = 55,36
SuTemp[4,12] = 57,28	SuTemp[4,12] = 57,11	SuTemp[4,12] = 56,90	SuTemp[4,12] = 56,64	SuTemp[4,12] = 56,30
SuTemp[5,12] = 58,39	SuTemp[5,12] = 58,08	SuTemp[5,12] = 57,70	SuTemp[5,12] = 57,22	SuTemp[5,12] = 56,62
SuTemp[6,12] = 59,08	SuTemp[6,12] = 58,58	SuTemp[6,12] = 57,97	SuTemp[6,12] = 57,22	SuTemp[6,12] = 56,30
SuTemp[7,12] = 59,31	SuTemp[7,12] = 58,58	SuTemp[7,12] = 57,70	SuTemp[7,12] = 56,64	SuTemp[7,12] = 55,36
SuTemp[8,12] = 59,08	SuTemp[8,12] = 58,08	SuTemp[8,12] = 56,90	SuTemp[8,12] = 55,51	SuTemp[8,12] = 53,89
SuTemp[9,12] = 58,39	SuTemp[9,12] = 57,11	SuTemp[9,12] = 55,63	SuTemp[9,12] = 53,94	SuTemp[9,12] = 52,04
SuTemp[10,12] = 57,28	SuTemp[10,12] = 55,73	SuTemp[10,12] = 53,98	SuTemp[10,12] = 52,05	SuTemp[10,12] = 53,59
SuTemp[11,12] = 55,80	SuTemp[11,12] = 54,01	SuTemp[11,12] = 52,06	SuTemp[11,12] = 53,95	
SuTemp[12,12] = 54,04	SuTemp[12,12] = 52,06	SuTemp[12,12] = 54,32		
SuTemp[13,12] = 52,07	SuTemp[13,12] = 54,68			
SuTemp[14,12] = 52,07

**Table 3:** Drying chamber area 20 m^2^.

*d* = 9	*d* = 8	*d* = 7	*d* = 6	*d* = 5
SuTemp[1,12] = 43,11	SuTemp[1,12] = 43,37	SuTemp[1,12] = 43,33	SuTemp[1,12] = 43,23	SuTemp[1,12] = 43,24
SuTemp[2,12] = 43,21	SuTemp[2,12] = 43,57	SuTemp[2,12] = 43,60	SuTemp[2,12] = 43,38	SuTemp[2,12] = 43,39
SuTemp[3,12] = 43,29	SuTemp[3,12] = 43,59	SuTemp[3,12] = 43,73	SuTemp[3,12] = 43,43	SuTemp[3,12] = 43,39
SuTemp[4,12] = 43,33	SuTemp[4,12] = 43,58	SuTemp[4,12] = 43,73	SuTemp[4,12] = 43,38	SuTemp[4,12] = 43,24
SuTemp[5,12] = 43,33	SuTemp[5,12] = 43,59	SuTemp[5,12] = 43,60	SuTemp[5,12] = 43,23	SuTemp[5,12] = 43,24
SuTemp[6,12] = 43,29	SuTemp[6,12] = 43,57	SuTemp[6,12] = 43,33	SuTemp[6,12] = 43,84	
SuTemp[7,12] = 43,21	SuTemp[7,12] = 43,37	SuTemp[7,12] = 43,41		
SuTemp[8,12] = 43,11	SuTemp[8,12] = 43,02			
SuTemp[9,12] = 43,84				

*d* = 14	*d* = 13	*d* = 12	*d* = 11	*d* = 10
SuTemp[1,12] = 43,19	SuTemp[1,12] = 43,17	SuTemp[1,12] = 43,24	SuTemp[1,12] = 43,09	SuTemp[1,12] = 43,10
SuTemp[2,12] = 43,38	SuTemp[2,12] = 43,33	SuTemp[2,12] = 43,45	SuTemp[2,12] = 43,19	SuTemp[2,12] = 43,19
SuTemp[3,12] = 43,52	SuTemp[3,12] = 43,46	SuTemp[3,12] = 43,62	SuTemp[3,12] = 43,26	SuTemp[3,12] = 43,26
SuTemp[4,12] = 43,64	SuTemp[4,12] = 43,55	SuTemp[4,12] = 43,74	SuTemp[4,12] = 43,32	SuTemp[4,12] = 43,31
SuTemp[5,12] = 43,71	SuTemp[5,12] = 43,61	SuTemp[5,12] = 43,80	SuTemp[5,12] = 43,34	SuTemp[5,12] = 43,33
SuTemp[6,12] = 43,75	SuTemp[6,12] = 43,63	SuTemp[6,12] = 43,82	SuTemp[6,12] = 43,34	SuTemp[6,12] = 43,31
SuTemp[7,12] = 43,76	SuTemp[7,12] = 43,63	SuTemp[7,12] = 43,80	SuTemp[7,12] = 43,32	SuTemp[7,12] = 43,26
SuTemp[8,12] = 43,75	SuTemp[8,12] = 43,61	SuTemp[8,12] = 43,74	SuTemp[8,12] = 43,26	SuTemp[8,12] = 43,19
SuTemp[9,12] = 43,71	SuTemp[9,12] = 43,55	SuTemp[9,12] = 43,62	SuTemp[9,12] = 43,19	SuTemp[9,12] = 43,10
SuTemp[10,12] = 43,64	SuTemp[10,12] = 43,46	SuTemp[10,12] = 43,45	SuTemp[10,12] = 43,09	SuTemp[10,12] = 43,82
SuTemp[11,12] = 43,52	SuTemp[11,12] = 43,33	SuTemp[11,12] = 43,24	SuTemp[11,12] = 43,93	
SuTemp[12,12] = 43,38	SuTemp[12,12] = 43,17	SuTemp[12,12] = 43,69		
SuTemp[13,12] = 43,19	SuTemp[13,12] = 43,70			
SuTemp[14,12] = 43,61

**Table 4:** Drying chamber area 30 m^2^.

*d* = 9	*d* = 8	*d* = 7	*d* = 6	*d* = 8
SuTemp[1,12] = 43,89	SuTemp[1,12] = 43,81	SuTemp[1,12] = 43,77	SuTemp[1,12] = 43,82	SuTemp[1,12] = 43,99
SuTemp[2,12] = 43,90	SuTemp[2,12] = 43,77	SuTemp[2,12] = 43,60	SuTemp[2,12] = 43,55	SuTemp[2,12] = 43,99
SuTemp[3,12] = 43,89	SuTemp[3,12] = 43,83	SuTemp[3,12] = 43,74	SuTemp[3,12] = 43,45	SuTemp[3,12] = 43,99
SuTemp[4,12] = 43,90	SuTemp[4,12] = 43,76	SuTemp[4,12] = 43,74	SuTemp[4,12] = 43,55	SuTemp[4,12] = 43,99
SuTemp[5,12] = 43,90	SuTemp[5,12] = 43,83	SuTemp[5,12] = 43,60	SuTemp[5,12] = 43,82	SuTemp[5,12] = 43,64
SuTemp[6,12] = 43,89	SuTemp[6,12] = 43,77	SuTemp[6,12] = 43,77	SuTemp[6,12] = 43,40	
SuTemp[7,12] = 43,90	SuTemp[7,12] = 43,81	SuTemp[7,12] = 43,28		
SuTemp[8,12] = 43,89	SuTemp[8,12] = 43,02			
SuTemp[9,12] = 43,82				

*d* = 14	*d* = 13	*d* = 12	*d* = 11	*d* = 10
SuTemp[1,12] = 47,35	SuTemp[1,12] = 16,27	SuTemp[1,890] = 43,19	SuTemp[1,12] = 56,09	SuTemp[1,12] = 43,99
SuTemp[2,12] = 47,47	SuTemp[2,12] = 16,34	SuTemp[2,890] = 43,21	SuTemp[2,12] = 56,10	SuTemp[2,12] = 46,00
SuTemp[3,12] = 47,32	SuTemp[3,12] = 16,25	SuTemp[3,890] = 18,18	SuTemp[3,12] = 56,09	SuTemp[3,12] = 43,99
SuTemp[4,12] = 43,44	SuTemp[4,12] = 43,34	SuTemp[4,890] = 18,22	SuTemp[4,12] = 56,10	SuTemp[4,12] = 56,00
SuTemp[5,12] = 43,39	SuTemp[5,12] = 43,26	SuTemp[5,890] = 46,17	SuTemp[5,12] = 56,09	SuTemp[5,12] = 53,99
SuTemp[6,12] = 43,36	SuTemp[6,12] = 43,31	SuTemp[6,890] = 43,22	SuTemp[6,12] = 56,09	SuTemp[6,12] = 54,00
SuTemp[7,12] = 43,44	SuTemp[7,12] = 43,31	SuTemp[7,890] = 43,17	SuTemp[7,12] = 56,10	SuTemp[7,12] = 53,99
SuTemp[8,12] = 43,36	SuTemp[8,12] = 43,26	SuTemp[8,890] = 43,22	SuTemp[8,12] = 56,09	SuTemp[8,12] = 45,00
SuTemp[9,12] = 43,39	SuTemp[9,12] = 46,34	SuTemp[9,890] = 43,18	SuTemp[9,12] = 56,10	SuTemp[9,12] = 43,99
SuTemp[10,12] = 43,44	SuTemp[10,12] = 46,25	SuTemp[10,890] = 46,2	SuTemp[10,12] = 46,09	SuTemp[10,12] = 43,91
SuTemp[11,12] = 43,32	SuTemp[11,12] = 46,34	SuTemp[11,890] = 43,19	SuTemp[11,12] = 43,87	
SuTemp[12,12] = 43,47	SuTemp[12,12] = 46,27	SuTemp[12,890] = 43,76		
SuTemp[13,12] = 43,35	SuTemp[13,12] = 43,58			
SuTemp[14,12] = 43,37				

Demonstrated results of computer modeling show that as bigger area higher capacity of heating elements. However, temperature still stays in stated interval. It’s important to note that as against using entire heating elements at the moment, using of impulse elements create saving of electricity average by 35 percent. To confirm the results of computer modeling and the conclusions drawn, we will conduct experimental studies in grain storage facilities located in the city of Kislovodsk, Stavropol Territory.

### Experimental researches

As an object of research, let's take a granary owned by a private entrepreneur. Address: Podkumok village, Stavropol Territory. Area of seed storage is 29,98 m^2^. Date of experiment is 1.07.2022. Outside temperature is 17 °C, it little rains. Stated temperature regime of dryer is 35–45 °C. Experimental results are in Table 5.

**Table 5:** Drying chamber area 30 m^2^.

*d* = 9	*d* = 8	*d* = 7	*d* = 6	*d* = 5
SuTemp[1,12] = 35,8	SuTemp[1,12] = 35,8	SuTemp[1,12] = 35,7	SuTemp[1,12] = 35,7	SuTemp[1,12] = 43,0
SuTemp[2,12] = 36,0	SuTemp[2,12] = 37,7	SuTemp[2,12] = 37,6	SuTemp[2,12] = 39,4	SuTemp[2,12] = 43,0
SuTemp[3,12] = 38,9	SuTemp[3,12] = 39,3	SuTemp[3,12] = 43,7	SuTemp[3,12] = 43,2	SuTemp[3,12] = 43,7
SuTemp[4,12] = 40,9	SuTemp[4,12] = 43,6	SuTemp[4,12] = 43,7	SuTemp[4,12] = 43,4	SuTemp[4,12] = 43,1
SuTemp[5,12] = 43,0	SuTemp[5,12] = 43,3	SuTemp[5,12] = 43,6	SuTemp[5,12] = 34,3	SuTemp[5,12] = 43,1
SuTemp[6,12] = 43,8	SuTemp[6,12] = 43,7	SuTemp[6,12] = 43,7	SuTemp[6,12] = 31,1	
SuTemp[7,12] = 43,0	SuTemp[7,12] = 43,8	SuTemp[7,12] = 43,2		
SuTemp[8,12] = 43,8	SuTemp[8,12] = 43,2			
SuTemp[9,12] = 43,8				

*d* = 14	*d* = 13	*d* = 12	*d* = 11	*d* = 10
SuTemp[1,12] = 37,5	SuTemp[1,12] = 16,2	SuTemp[1,890] = 28,1	SuTemp[1,12] = 56,0	SuTemp[1,12] = 43,9
SuTemp[2,12] = 37,4	SuTemp[2,12] = 26,4	SuTemp[2,890] = 32,2	SuTemp[2,12] = 56,1	SuTemp[2,12] = 46,0
SuTemp[3,12] = 37,2	SuTemp[3,12] = 46,2	SuTemp[3,890] = 38,1	SuTemp[3,12] = 56,0	SuTemp[3,12] = 43,9
SuTemp[4,12] = 33,4	SuTemp[4,12] = 43,2	SuTemp[4,890] = 38,2	SuTemp[4,12] = 56,1	SuTemp[4,12] = 56,0
SuTemp[5,12] = 43,3	SuTemp[5,12] = 43,8	SuTemp[5,890] = 46,1	SuTemp[5,12] = 56,0	SuTemp[5,12] = 53,9
SuTemp[6,12] = 43,3	SuTemp[6,12] = 43,4	SuTemp[6,890] = 43,2	SuTemp[6,12] = 56,0	SuTemp[6,12] = 54,0
SuTemp[7,12] = 43,7	SuTemp[7,12] = 43,3	SuTemp[7,890] = 43,1	SuTemp[7,12] = 56,1	SuTemp[7,12] = 53,9
SuTemp[8,12] = 43,6	SuTemp[8,12] = 43,4	SuTemp[8,890] = 43,2	SuTemp[8,12] = 56,0	SuTemp[8,12] = 45,0
SuTemp[9,12] = 43,3	SuTemp[9,12] = 46,4	SuTemp[9,890] = 43,1	SuTemp[9,12] = 56,1	SuTemp[9,12] = 43,9
SuTemp[10,12] = 43,4	SuTemp[10,12] = 46,5	SuTemp[10,890] = 44,2	SuTemp[10,12] = 46,0	SuTemp[10,12] = 43,9
SuTemp[11,12] = 43,2	SuTemp[11,12] = 44,4	SuTemp[11,890] = 43,1	SuTemp[11,12] = 43,8	
SuTemp[12,12] = 45,7	SuTemp[12,12] = 44,7	SuTemp[12,890] = 44,7		
SuTemp[13,12] = 43,5	SuTemp[13,12] = 43,5			
SuTemp[14,12] = 43,3				

The results of the experiment confirm the possibility of replacing solid heating elements with impulse ones with their location in sections at an equidistant distance from each other. It is also important to note that there is a drop in temperature (in this place there is an entrance for vehicles), which the developed system can hardly cope with in the northern part of the storage. In the experiment with 11 and 10 heating elements, this caused an increase to a temperature of 56 °C, which is not critical, since grain drying is usually carried out at temperatures up to 110 °C.

## Discussion

This work is devoted to improving the economic efficiency of grain drying chambers by developing an optimal temperature field control system. Economic efficiency is achieved by replacing the existing permanent heating elements with pulsed ones with a relay control principle. The technological process of obtaining biodiesel fuel from vegetable raw materials is considered as an object of research.

Obtaining environmentally friendly fuels is a promising and high-tech direction. Due to the use of modern technologies, the cost of the final product is steadily decreasing. At the same time, there are quite a large number of unsolved problems. One of these tasks is the preparation and storage of raw materials. Often up to 15% of the oil suitable for processing is lost during drying and transportation. Having analyzed the world experience in this field, the team of authors suggests a more efficient way of drying and storing raw materials based on the theory of systems with distributed parameters and the Green function. After conducting computer modeling and comparing it with the results of experimental studies, the authors conclude that the cost of raw material preparation is reduced by 10–15%. At the same time, they note the presence of a more homogeneous thermal field, which contributes to the preservation of more oils ready for processing.

It is important to note that the obtained mathematical models and experimental results showed that using solid heating elements causes excessive waste of energy resources. So, for example, results of tables show, that there is a significant increase in power at the initial stage of operation of the heating elements. But at the end of the heating stage of the chamber, part of the heating elements is turned off. And it turns on only if the temperature drops. Standard solid heating elements are not capable of partial shutdown. In the above experiments, less than half of the heaters were involved. However, it is important to note that in the process of replenishing the barn, when an additional part of the grain was poured into the room, boundary conditions were violated and all heating elements were involved. As shown in^40,43–45^, the long-term operation of such systems, the payback of these projects comes much earlier.

It should be noted that the use of the obtained technology is limited by climatic conditions. As shown in^46–55^, the industrial application of some technologies is very limited at low temperatures.

According to the results of the six-month operation of this technology, electricity costs decreased by 33%. The load on the electrical network decreased by an average of 12%. According to the results of an independent examination, Protocol No. 4 of 04/15/2023, the percentage of grain whose quality was assessed as "unsatisfactory" was 2.21%. In 2021, the result of a similar examination was 11.6%.

## Conclusion

In the context of a reduction in the extraction of minerals, the issue of the use of renewable energy sources is acute. One of these sources includes biodiesel. Within the framework of this study, the initial stage of obtaining biodiesel fuel—the storage stage of raw materials is considered. The authors have identified existing production problems and, based on world experience, have proposed a number of the following technical solutions:

1. The peculiarity of these heating elements is the short-term supply of electrical energy to bring the heater into temperature mode. In the future, the power supply stops, turning on for short intervals when the heating element cools down below the set value. By reducing the time, electric energy is saved and, as a result, the cost is reduced.
2. Implementation of a room temperature control system. The proposed control system based on the Green function makes it possible to form a uniform temperature field over the entire drying area, preventing the formation of overheating or underheating zones, which is the main cause of oil loss, grain clumping, mold appearance, etc.

The paper also solves a number of scientific problems that are important for the analysis of temperature fields in the field of spatially distributed control systems, the main of which is the modernization of the classical Green function for solving problems in the field of three-dimensional control systems. The resulting equation makes it possible to describe with greater accuracy the temperature fields for which there is a solution to the problem of thermal conductivity under zero boundary conditions.

Thus, the authors have solved a wide range of tasks related to increasing the economic efficiency of the drying process of raw materials intended for biofuel production.

## Data Availability

The datasets generated and analysed during the current study are available in the drive.google.com repository, https://drive.google.com/drive/folders/1P_QnSW2EGHkrzaUhEM2Ln0dgonGfIBTs?usp=sharing.
